# Emerging Trends in Mid-Range Nursing Theories: A Scoping Review

**DOI:** 10.3390/nursrep15110382

**Published:** 2025-10-28

**Authors:** David Sancho-Cantus, Dolores Escrivá-Peiró, Cristina Cunha-Pérez

**Affiliations:** 1 Department of Nursing, Catholic University San Vicente Mártir, 46001 Valencia, Spain; escriva_dolpei@gva.es (D.E.-P.); cristina.cunha@ucv.es (C.C.-P.); 2 Intensive Care Unit, La Fe Polytechnic and University Hospital, 46026 Valencia, Spain

**Keywords:** nursing theories, nursing models, middle-range nursing theories, conceptual frameworks, theory development

## Abstract

**Background**: Nursing research has evolved through different historical stages, from the initial development of theoretical models to today’s challenges involving advanced practice and emerging technologies. Within this context, Middle-Range Nursing Theories (MRNTs) play a crucial role as a bridge between abstract conceptual frameworks and clinical practice. However, their recent production appears limited. **Aims**: To identify MRNTs published in the last five years, determine the main thematic fields addressed, and analyze current trends in their development. **Methods**: A scoping review was conducted in accordance with PRISMA-ScR guidelines. Databases searched included MEDLINE, CINAHL, PsycINFO, EMBASE, and Education Research Complete (August 2025). Eligible studies were published within the last five years in journals indexed in the *Journal Citation Reports* and explicitly proposed an MRNT. Exclusion criteria encompassed non-nursing theories, secondary applications of existing models, and purely methodological studies. **Results**: From 1230 initial records, 18 articles met the inclusion criteria. The *Revista Brasileira de Enfermagem* accounted for the highest number of publications. The identified MRNTs predominantly addressed clinical diagnoses and phenomena such as heart failure self-care, overweight, occupational stress, peripheral tissue perfusion, and social support networks. Most theories were derived from established nursing models (Orem, Roy, Levine, Neuman, Watson). Despite thematic diversity, few MRNTs had undergone methodological validation. **Conclusions**: Recent MRNT development remains limited and geographically concentrated, with Brazil emerging as a leading contributor. Strengthening methodological validation, clinical integration, and international dissemination is essential, as MRNTs continue to be pivotal tools for advancing nursing science, reinforcing disciplinary identity, and reducing the persistent gap between theory and practice.

## 1. Introduction

Nursing research is intrinsically linked to the evolution and development of the discipline itself. Throughout its history, several stages have been identified in the literature, each characterized by prevailing research trends and distinct scientific contributions:

### 1.1. Stage I (1977–1990): The Onset of Clinical Research and Theoretical Development [1]

During this period, nursing research emerged as a scientific discipline. Key topics included three main areas. First, the focus was on theoretical foundations and conceptual models. This involved developing and validating nursing theories from figures like Orem, Roy, and Rogers to legitimize the discipline. Second, clinical practice research concentrated on areas such as pain, nutrition, and pressure ulcer prevention. Finally, mental healthcare research was marked by a gradual paradigm shift from the biomedical model to a biopsychosocial approach. Issues such as stress and anxiety management and nursing roles in supporting patients with mental illness gained particular attention.

### 1.2. Stage II (1990–2000): The Rise in Evidence-Based Research [2]

This decade witnessed a transition from theoretical validation to the practical application of scientific evidence. Research priorities included improving care quality and patient safety, managing chronic diseases—especially in the context of Acquired Immune Deficiency Syndrome (AIDS)—and expanding community-based and primary healthcare initiatives in countries such as Spain.

### 1.3. Stage III (2000–2010): Globalization of Research and Multidisciplinary Approaches [3]

With the advent of internet access and enhanced databases, nursing research became increasingly globalized. This shift facilitated international collaboration through information and communication technologies (ICT). Furthermore, this stage marked a growing focus on aging populations, a response to the demographic trend towards progressively older societies.

### 1.4. Stage IV (2010–Nowadays): Advanced Practice Nursing and Contemporary Challenges [4]

The last decade has been characterized by greater methodological sophistication and a focus on global health challenges. This period has seen the inclusion of artificial intelligence and various forms of simulation in teaching. Moreover, it has involved the adoption of innovative methodologies aligned with ongoing technological and social transformations.

Nursing research provides substantial benefits when guided by established theoretical frameworks, as nursing theories provide a solid foundation for clinical decision-making grounded in the best available scientific evidence [5]. Evidence-based practice reduces variability in care delivery, which enhances quality and patient outcomes. It also improves patient satisfaction and can decrease healthcare costs [6].

### 1.5. Mid-Range Nurse Theories in Nursing (MRNT)

Since the publication of Middle-Range Theories: Application to Nursing Research and Practice [7], many scholars have expressed concern regarding the diminishing emphasis on nursing theory in education, practice, and research [8,9,10]. Gardner [11] stressed the need for professionals to master at least one distinctive way of reasoning inherent to their field, while Nelson [12] argued that theories and conceptual frameworks enable a deeper understanding of nursing practice. Likewise, the American Association of Colleges of Nursing [13], through its DNP Essentials document, highlights the relevance of science and theory in nursing education and practice, and these conclusions are supported by numerous studies, particularly from Brazil. In these studies, institutions of higher education were often the primary source of information related to MRNT.

Nursing theories, including MRNTs, are thus essential to advancing the discipline. They provide a conceptual basis to guide practice, shape research, and transform interdisciplinary knowledge into a nursing-specific perspective [14].

In the social sciences, MRNTs emerged in response to the challenge of constructing a unified theory capable of explaining all aspects of social behavior, organization, and change. Rather than proposing an all-encompassing framework, these theories focus on well-defined phenomena. This targeted approach allows them to produce rigorous explanations of particular social components. Positioned between abstract grand theories and empirical research, MRNTs allow for the development of testable hypotheses, making them valuable methodological tools for advancing disciplinary knowledge [15].

A recent editorial in *Aquichan* (Colombia) reported that across the Americas (United States, Canada, Brazil, and Central and South America), 402 publications were indexed between 2018 and 2022 using the descriptor “Nursing Theory.” Building on this evidence, the present review aims to examine recent global contributions to nursing research, specifically regarding the development of MRNTs.

The following research questions guided this review:

(a) How many MRNT have been published in nursing over the last 5 years?
(b) Which fields or topics are most frequently addressed within these theories?
(c) What current trends characterize research on MRNTs?

## 2. Materials and Methods

### 2.1. Design

This scoping review (ScR) was conducted in accordance with the *Preferred Reporting Items for Systematic Reviews and Meta-Analyses* extension for scoping reviews (PRISMA-ScR) [16]. ScRs are widely employed to synthesize research evidence. They are particularly well suited to mapping the existing body of literature within a specific field in terms of its scope, content, and volume [17,18]. For this reason, ScRs are often called “mapping reviews” [19], as they aim to identify and synthesize key concepts underpinning a research area [20]. Therefore, the primary objective of this review was not to provide an in-depth analysis of the theory construction process. Instead, it aimed to map the thematic areas in which such theories have been developed.

### 2.2. Eligibility Criteria

Both quantitative and qualitative research designs were initially considered for inclusion. Quantitative designs encompassed observational studies (e.g., descriptive, cohort, cross-sectional, case studies, and case series) and experimental designs such as clinical trials. Qualitative designs included methodologies such as phenomenology, grounded theory, ethnography, and participatory action research. Studies were eligible if they explicitly proposed a middle-range nursing theory (MRNT) and were published within the last five years. An additional requirement was publication in a journal indexed in the *Journal Citation Reports.* This indexing system was chosen because it serves as a benchmark for journal relevance, visibility, and quality across disciplines [21]. The exclusion criteria were as follows: theories not focused on nursing, studies lacking objective quality indicators (e.g., doctoral theses), applications of existing theoretical models without new theoretical development, and purely methodological studies that did not propose an MRNT. Only studies published in English were included, given its status as the predominant language in academic publishing.

### 2.3. Search Strategy and Information Sources

The search strategy followed a three-step process, in line with PRISMA-ScR methodology and the Joanna Briggs Institute guidelines [22,23]. First, an initial search was performed, analyzing keywords from titles, abstracts, and article descriptors. Second, a comprehensive search was conducted across all included databases using all identified keywords and index terms. Third, the reference lists of all identified reports and articles were screened for additional relevant studies. This search was carried out by two independent reviewers, one of whom specialized in bibliometrics and documentation. The databases consulted included MEDLINE, CINAHL, PsycINFO, EMBASE, and Education Research Complete, accessed via the EBSCO platform. The initial keywords used were nursing models and theoretical. The searches were conducted between July and August 2025.

### 2.4. Data Extraction Process

Data extraction was performed using an expanded version of the Joanna Briggs Institute Meta-Analysis Statistics Review and Assessment Instrument (JBI-MAStARI) [24]. Duplicates were removed using the Rayyan reference management tool [25]. Initially, three reviewers independently screened titles and abstracts to exclude irrelevant studies. The full texts of the remaining articles were then retrieved for analysis. Subsequently, each article was reviewed independently by two researchers. Any discrepancies were resolved through discussion; if a consensus could not be reached, the principal investigator made the final decision. In this review, no such disagreements were recorded.

### 2.5. Critical Appraisal

No critical appraisal of the included studies was undertaken, as the aim of this review was not to assess clinical outcomes or intervention effectiveness but rather to map the existing evidence on the topic.

### 2.6. Data Analysis and Synthesis of Results

The extracted data were compiled into a Microsoft Excel table [26], which was then manually reviewed to ensure accuracy. This table included the author(s), year of publication, article title, journal, and main findings. Finally, the information was synthesized into a summary table, and the analyzed variables were presented graphically in accordance with the methodological guidelines of the Joanna Briggs Institute [27] (See Table 1).

## 3. Results

**Figure 1:**
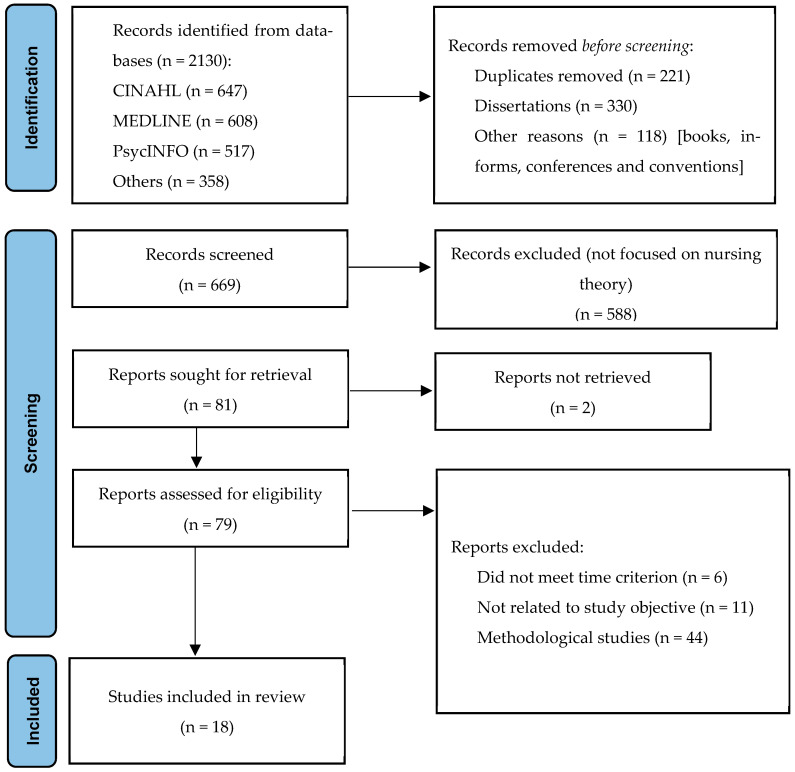
Preferred Reporting Items for Systematic Reviews and Meta-Analyses (PRISMA-ScR) flow diagram illustrating the screening and selection process for the scoping review [51].

The initial literature search identified 1230 records. After removing duplicates and other ineligible texts as described in the previous section, 669 records remained. Following the title and abstract screening, 588 records were excluded, leaving 81 studies for full-text review. Of these, 18 articles met all inclusion criteria and were ultimately included in the analysis. The entire selection and screening process is presented in a PRISMA flow diagram (Figure 1).

### Characteristics of Sources of Evidence

**Figure 2:**
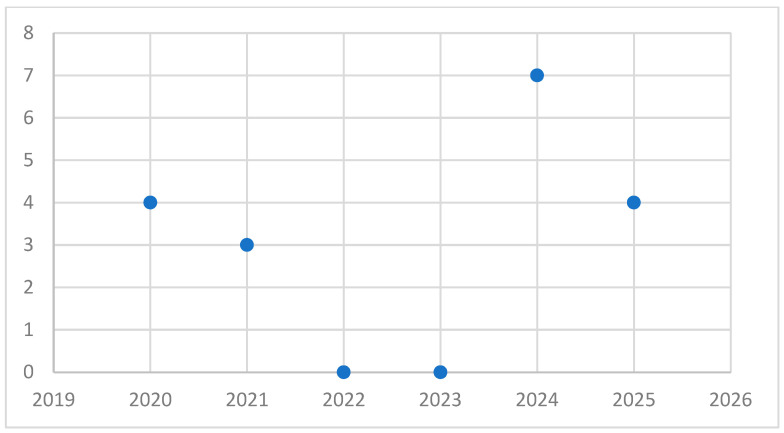
Number of publications by year.

The included studies spanned a five-years period. The year 2024 was the most productive, with seven publications (38.8%) (Figure 2).

**Figure 3:**
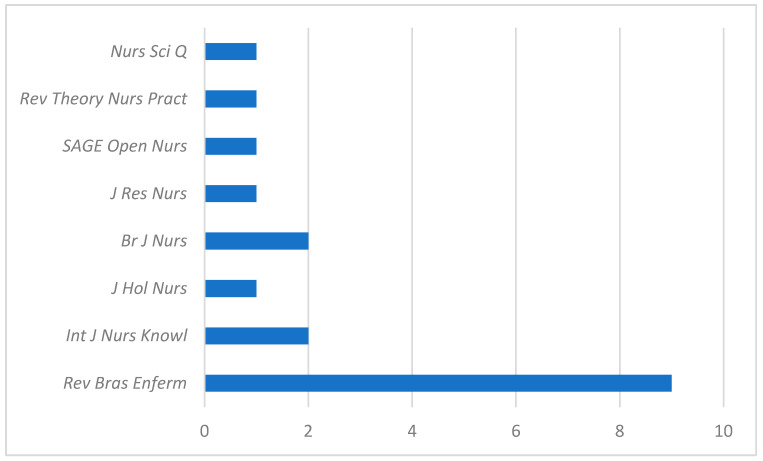
Journals in which MRNTs have been published.

Regarding the publication venues, the *Revista Brasileira de Enfermagem* was the leading Journal with eight articles. The *International Journal of Nursing Knowledge* published two of the included studies, and the remaining articles were distributed across various other journals (Figure 3).

The following middle-range nursing theories (MRNTs) were published during the study period, along with their objectives and notable methodological approaches.

Middle-Range Theory for the Nursing Diagnosis of Low Health Self-Efficacy [28]: This theory, which is based on Bandura’s social cognitive theory [29], addresses low health self-efficacy. Its objective was to validate a diagnostic construct for potential incorporation into North American Nursing Diagnosis Association (NANDA) International (NANDA-I). Such a construct would serve as a useful tool for nursing professionals in the early detection of personal inefficacy.

Mid-Range Theory for the Nursing Diagnosis of Excessive Fluid Volume in Pregnant Women [31]: This MNRT was developed to understand the causal mechanisms and clinical consequences of the nursing diagnosis “Excessive Fluid Volume” (00026) in pregnant women. The theory integrates both physiological and pathological conditions to explain fluid retention and its clinical indicators. In doing so, it offers insights into the interactions between causative factors and clinical manifestations.

Mid-range Theory of Wound Pruritus [31]: This theory provides a theoretical foundation for studying wound-related itching (pruritus), a problem often overlooked in practice yet highly relevant to patients’ quality of life. Based on Levine’s conservation model [32], it conceptualizes wound itching as a subjective phenomenon. This phenomenon may be intermittent or persistent, can range from mildly annoying to severely distressing, and is common in individuals with impaired skin integrity. Understanding these aspects could support nurses in delivering interventions to alleviate discomfort.

Development of a medium-range theory on self-care in heart failure [33]: Drawing on Orem’s self-care theory [34], this MRNT adapts self-care determinants to the context of patients with heart failure. The theory emphasizes the roles of healthcare systems, individual characteristics, and the concept of self-care agency. Within this concept of agency, nurses guide patients to achieve independent and autonomous self-care. This approach ultimately aims to enhance clinical nursing outcomes.

Poor Knowledge in Individuals with Heart Failure, a middle-range nursing theory [35]: Based on Roy’s Adaptation Model, this theory identified two attributes, eight antecedents, and seven consequences associated with inadequate knowledge in heart failure patients. This framework enables nurses to structure individualized interventions and improve clinical judgment.

**Figure 4:**
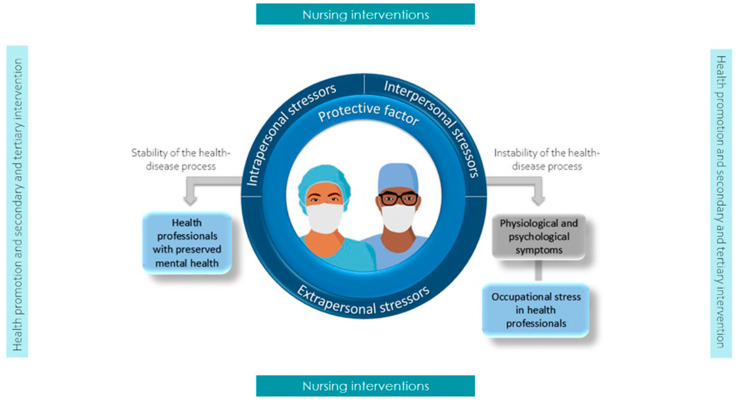
Diagram of the MNRT of occupational stress in health professionals [36].

Middle-Range Theory of Occupational Stress in Health Professionals [36]: Developed from Betty Neuman’s systems model, this MRNT proposes a nursing diagnosis for occupational stress. The theory integrates intra-, inter-, and extra-personal stressors. It also considers protective factors that influence nurses’ responses to these work-related stressors (Figure 4).

**Figure 5:**
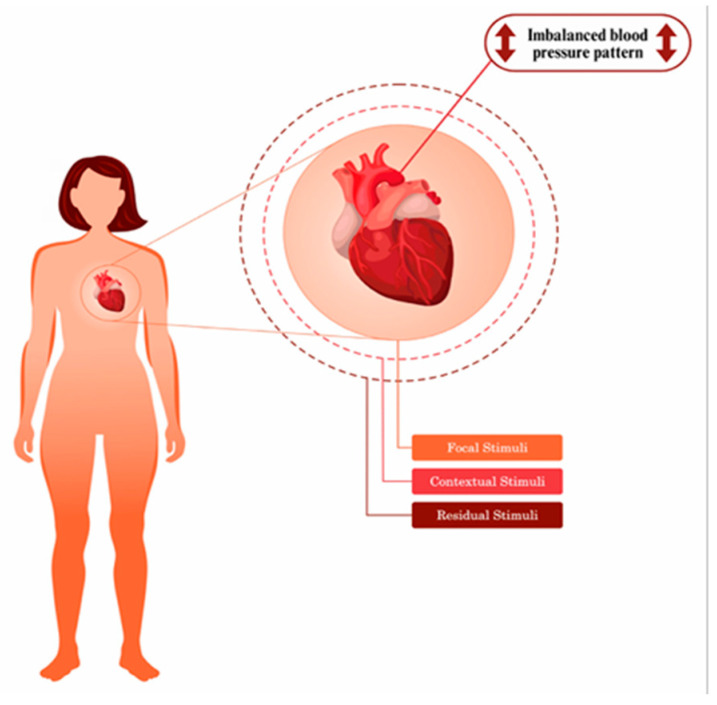
Pictorial diagram of risk for imbalanced blood pressure pattern [37].

Middle-Range Theory for the Risk of Imbalanced Blood Pressure Patterns in Incarcerated Women [37]: This theory examines variables associated with imbalanced blood pressure patterns in incarcerated women, aiming to establish the theoretical–causal validity of the nursing diagnosis *Risk of Unstable Blood Pressure* within this specific population. When analyzing elevated blood pressure in this group, the theory distinguishes between three types of factors: focal or stimuli (e.g., sedentary lifestyle, high-calorie diet), contextual (e.g., insomnia, excess weight), and residual (e.g., sex, family history) (Figure 5).

**Figure 6:**
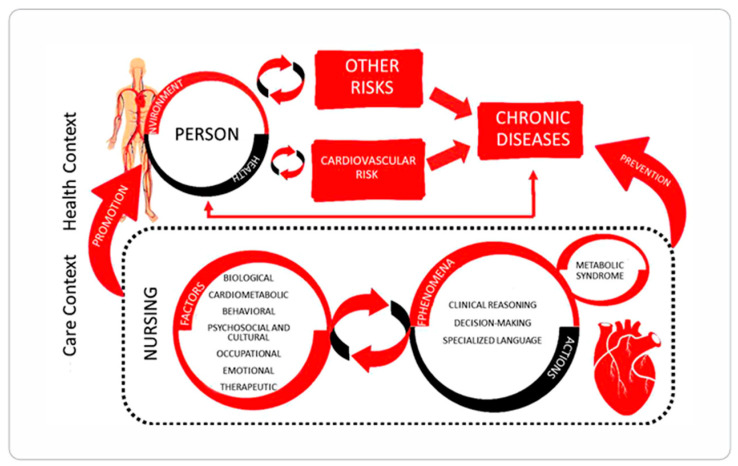
Modeling the concepts of Theory of Care within the context of cardiovascular risk nursing metaparadigm [38].

Middle-Range Theory of Nursing for Care in the Context of Cardiovascular Risk (TEORISC) [38]: This descriptive MRNT aims to characterize care practices and prescribe interventions designed to promote health and reduce cardiovascular risk. It was developed through research induction and practice standards, using the CIPE^®^ (International Classification for Nursing Practice) terminology subset. The theory highlights that care strategies must consider both risk factors and context-related phenomena. This ensures the interventions align with the patient’s vital needs. Furthermore, the approach transcends purely biological or technical dimensions by maintaining a focus on the human factor (Figure 6).

Middle-Range Theory of Nursing Diagnosis of Sedentary Lifestyle in Young Adults [39]: Based on Roy’s Adaptation Model [40], this predictive MRNT identifies clinical variables associated with sedentary behavior in young adults. The theory emphasizes how sedentary lifestyles and their consequences are influenced by personal and social characteristics, physical inactivity, and insufficient levels of recommended physical activity.

Medium-Range Theory of Nursing Diagnosis of Overweight [41]: This MRNT four distinct categories of contributing factors. Triggering factors include a sedentary lifestyle, unhealthy eating habits, and poor sleep quality. Predisposing factors consist of emotional disorders, personal or family history of overweight, and an active academic relationship. Incapacitating factors are alcoholism and the use of obesogenic medications, while reinforcing factors include early menarche, low family income, and female sex.

Construction of an MRNT for transpersonal home care [42]: Developed from Jean Watson’s *Theory of Human Care/Unitary Caring Science* [43], this MRNT incorporates concepts, assumptions, and propositions for home-based nursing care. It is designed to be applicable to patients, caregivers, and families in diverse social, cultural, political, and economic contexts.

Mid-range theory for the nursing phenomenon of ineffective social support network [44]: This theory identifies the etiological factors and clinical indicators that underlie ineffective social support networks. It addresses several domains, including individual characteristics, the network’s members and configuration, and external contextual influences.

Advances in Nursing Science—Development of a middle-range theory for research on systemic communication in complex hospital environments [45]: This MRNT emphasizes the critical role of communication within complex healthcare settings. It draws on a wide range of theoretical sources, including the Effective Communication Framework among Nurses, Symbolic Interactionism, Information Theory, Gerbner’s Communication Model, and Complexity Theory.

Middle-range theory for the nursing diagnosis of dysfunctional response to ventilatory weaning [46]: This MRNT aims to improve knowledge about the process of discontinuing mechanical ventilation. The ultimate goal is to enhance nursing care delivery during ventilatory weaning.

Middle-range theory on ineffective peripheral tissue perfusion in patients with diabetic foot—a mid-range theory [47]: This theory expands conceptual understanding of the nursing on ineffective peripheral tissue perfusion by identifying its causal relationships. This approach aims to minimize knowledge gaps and guide evidence-based nursing interventions for patients with diabetic foot.

Introduction to Rivera’s Gender Affirming Nursing Care Model—A Middle-Range Theory [48]: This model was tested as a tool to foster gender-neutral thinking in nursing practice. It supports nurses in identifying implicit and explicit biases through self-reflection and personal growth strategies. By encouraging such paradigm shifts, the model facilitates the development of gender-affirming nursing practice. However, the authors recommend further evaluation of its impact on patient outcomes.

The Person-centered Nursing Framework—a mid-range theory for nursing practice [49]: This discussion paper presents the theoretical foundations of the Person-Centered Nursing Framework (PCNF). It also explains the framework unique characteristics and demonstrates its applicability as a guiding middle-range theory for nursing practice.

The middle-range theory of the nursing diagnosis “ineffective health self-management” [50]: This theory was designed specifically for patients living with HIV. It provides a structured approach to support nurses in making clinical judgments about ineffective health self-management. Ultimately, its goal is to improve assessment and care planning for this population.

## 4. Discussion

Research and its resulting scientific output are essential for the development and consolidation of any discipline. This is particularly true in clinical fields like nursing, where the scope extends beyond healthcare to include education and management. This diversity makes nursing a dynamic domain where research plays a pivotal role in supporting evidence-based practice, ensuring quality of care, and fostering innovation in healthcare [18,25]. Historically, nursing research has focused on elderly care, end-of-life issues, the health effects of stress, and chronic disease management. A cross-cutting theme has been self-care and pedagogical strategies for its teaching. These topics reflect both responses to emerging social and epidemiological needs and the growing interest in strengthening patient autonomy. Within this framework, middle-range nursing theories (MRNTs) are crucial, as they bridge the gap between abstract grand theories and real-world nursing practice. Their development gained momentum in the 1980s. Pioneering examples, such as the *Theory of Obstetric Nursing Care*, demonstrated how this intermediate conceptualization could facilitate the translation of theoretical knowledge into clinical and educational settings [52,53].

Despite the exponential growth of scientific publications in nursing since 2006, the development of new MRNTs has been limited in recent years. This situation may be attributable to the ongoing debate about the role of theory within nursing. Some authors argue that an excessive focus on clinical practice has relegated theoretical construction. In contrast, others contend that a robust conceptual framework is essential for establishing a distinctive disciplinary identity. Consequently, the scarcity of new MRNTs can be interpreted in two ways: as a simple production gap, and as a reflection of an unresolved epistemological debate [54].

A recent bibliometric analysis revealed that, in the Brazilian context, MRNTs represent the most developed type of nursing theory, accounting for 82.1% of all identified proposals, with a significant increase in output since 2018 [55]. This finding suggests that the production of MRNTs may be experiencing a resurgence in some regions, possibly driven by research incentive policies and the consolidation of academic groups. Nonetheless, significant challenges remain. These include balancing theory with practice, achieving greater disciplinary recognition, and enhancing international dissemination. Overcoming these hurdles indicates that the global development of MRNTs will require sustained, coordinated efforts.

### 4.1. Limitations

A key limitation of this review was the scarcity of recently published MRNTs, with even fewer studies providing methodological validation [56]. Additional limitations include the following:

First, the restriction of sources to English- language articles indexed in the *Journal Citation Reports* (JCR) represents a significant limitation. While the criterion ensured a baseline of quality and visibility, it also introduces potential bias. Consequently, our findings likely underestimated the total production of MRNTs by excluding relevant publications from non-JCR journals or those published in other languages, such as Spanish and Portuguese. This is particularly noteworthy given the significant contribution from countries like Brazil.

Second, the exclusion of grey literature, such as doctoral theses, reports, and conference proceedings, is another limitation. This decision means the review did not capture emerging MRNTs or those in the early stages of development. As a result, the findings are skewed towards theories that have already reached the final stages of publication.

Third, in line with scoping review methodology, a critical appraisal of the quality or rigor of the included studies was not performed. Therefore, the conclusions are limited to mapping the thematic areas and volume of research. They do not extend to an assessment of the methodological validity of MRNTs themselves.

Time period: The restriction to the last five years fulfilled the objective of identifying emerging trends, but excluded influential theories published immediately prior to this period that could be the basis for recent production.

### 4.2. Future Directions

Future research should prioritize the methodological validation of existing MRNTs. Such validation is necessary to assess their quality, generalizability, and applicability across diverse clinical contexts. The current concentration of MRNT development in Brazil, a country with a strong theoretical tradition, highlights a clear need. Specifically, theoretical development must be encouraged in other regions, particularly within the English-speaking academic world. To promote this activity globally, increased funding and editorial support will be essential.

To ensure that MRNTs are tools of global value, their current geographical concentration and lack of validation must be overcome. To achieve this, it is crucial for academic institutions and funding bodies to promote the formation of multinational research consortia. These consortia could then empirically and interculturally test existing theories. This primary strategy should be complemented by two additional policies. First, theoretical validation should be integrated into postgraduate training. Second, multilingual publishing agreements should be established to ensure these conceptual frameworks are disseminated and applied in diverse health systems. Together, these actions are essential for ensuring the scientific and practical relevance of nursing worldwide.

## 5. Conclusions

This scoping review reveals a key paradox: while overall scientific production in nursing has grown substantially, the development of middle-range nursing theories (MRNTs) remains limited. Furthermore, this development is often confined to specific regional contexts, particularly Brazil. Over the past five years, several MRNTs have been focused on specific clinical phenomena, such as self-care in heart failure and occupational stress. This focus highlights their vital role as conceptual bridges between abstract grand theories and concrete clinical practice.

Nevertheless, significant challenges persist. The most critical among these are the need for rigorous methodological validation and the effective integration of these theories into clinical practice. The issue is rooted in a long-standing tension between clinical practice priorities and the demands of theoretical development, a conflict that continues to hinder the establishment of a cohesive disciplinary foundation.

To address this gap, it is imperative to strengthen the methodological validation of existing MRNTs and foster their incorporation into both clinical and educational settings. Furthermore, promoting international collaboration and publication in high-impact journals will be essential. These actions are needed to facilitate the dissemination of MRNTs beyond local contexts and, ultimately, to contribute to a global, unified vision of nursing science.

In essence, MRNTs are indispensable tools for advancing nursing knowledge. They offer intermediate frameworks that guide research, practice, and education from a perspective consistent with the discipline’s identity. Their continued development and application will be key to addressing contemporary healthcare challenges and ensuring the delivery of high-quality, evidence-based care. Looking forward, there is a clear need for research to focus on two areas: the rigorous validation of existing theories and the construction of new MRNTs that address modern challenges such as advanced practice, artificial intelligence, and telehealth.

## Figures and Tables

**Table 1:** Summary of key findings.

Title	Authors	Journal	Main Findings
Middle-Range Theory for the Nursing Diagnosis of Low Health Self-Efficacy [28,29]	Barreiro et al.	*Rev Bras Enferm* ^4^	The key concepts underlying the new nursing diagnosis of low health self-efficacy were identified and defined.
Middle-Range Theory for the Nursing Diagnosis of Excessive Fluid Volume in Pregnant Women [30]	Fróes et al.	*Rev Bras Enferm* ^4^	Incorporated physiological and pathological factors to explain excessive fluid volume, improving understanding of causal factors and clinical indicators.
Development of the Middle-Range Theory of Wound Pruritus [31,32]	Paul J.	*Br J Nurs* ^2^	Outlined the development of the wound pruritus theory based on Levine’s conservation model, integrating its concepts and propositions.
Development of a Middle-Range Theory of Heart Failure Self-Care [33,34]	Attallah et al.	*Nurs Sci Q* ^8^	Described the strategy for constructing a middle-range theory addressing self-care in heart failure.
Impaired Knowledge in Individuals with Heart Failure: A Middle-Range Nursing Theory [35]	Da Silva, et al.	*Rev Bras Enferm* ^4^	Provided a framework to guide nurses’ clinical judgment regarding knowledge deterioration in heart failure, supporting individualized interventions to improve quality of life.
Middle-Range Theory of Occupational Stress in Health Professionals [36]	Costa et al.	*SAGE Open Nurs* ^6^	Supported the development of elements for proposing a nursing diagnosis of occupational stress, strengthening diagnostic reasoning and guiding preventive interventions.
Risk for Imbalanced Blood Pressure Pattern among Incarcerated Women: Middle-Range Theory [37]	Da Silva et al.	*Rev Bras Enferm* ^4^	Provided a theoretical basis for understanding the risk of imbalanced blood pressure in incarcerated women, aiding in planning cardiovascular health interventions.
Middle-Range Theory for Nursing Care in the Context of Cardiovascular Risk [38]	Félix et al.	*Rev Bras Enferm* ^4^	Contributed to developing knowledge that supports nursing care planning for individuals at cardiovascular risk.
Middle-Range Theory of the Nursing Diagnosis of Sedentary Lifestyle in Young Adults [39,40]	Fernandes et al.	*Rev Bras Enferm* ^4^	Identified determinants of sedentary lifestyle in young adults to support evidence-based nursing interventions.
Middle-Range Theory of the Nursing Diagnosis: Overweight [41]	Costa et al.	*Rev Bras Enferm* ^4^	Enhanced understanding of the nursing diagnosis of overweight in adolescents and young adults by identifying causal and predisposing factors.
Construction of a Middle-Range Nursing Theory for Transpersonal Home Care [42,43]	Tonin et al.	*Rev Bras Enferm* ^4^	Developed a theoretical model for home care addressing physiological, psychological, sociocultural, developmental, and spiritual needs; pending empirical validation.
Middle-Range Theory for the Nursing Phenomenon of Ineffective Social Support Networks [44]	De França et al.	*Int J Nurs Knowl* ^5^	Provided an understanding of individuals from an interpersonal perspective, highlighting how networks of virtues and vices influence health outcomes negatively.
Advancing Nursing Science: Deriving a Middle-Range Theory for System-Level Communication Research in Complex Hospital Environments [45]	Brittain et al.	*Rev Theory Nurs Pract* ^7^	Addressed the need to optimize healthcare communication research through robust theoretical frameworks, emphasizing the complexity of hospital settings.
Middle-Range Theory for the Nursing Diagnosis of Dysfunctional Ventilatory Weaning Response [46]	Lemo et al.	*Int J Nurs Knowl* ^5^	Applied utility theory to nursing care during the ventilatory weaning process to enhance clinical decision-making.
Middle-Range Theory on Ineffective Peripheral Tissue Perfusion in Patients with Diabetic Foot [47]	Moraes et al.	*Rev Bras Enferm* ^4^	Expanded conceptual understanding and demonstrated causal relationships among factors involved in ineffective peripheral tissue perfusion.
Introduction to Rivera’s Gender Affirming Nursing Care Model: A Middle-Range Theory [48]	Rivera D., et al.	*J Holist Nurs* ^1^	Introduced a framework integrating relationship, knowledge, and commitment domains to guide gender-affirming nursing care practices
The Person-Centered Nursing Framework: A Middle-Range Theory for Nursing Practice [49]	McCance et al.	*J Res Nurs* ^3^	Highlights the contextual, attitudinal, and moral dimensions of humanitarian nursing care practices.
Middle-Range Theory of the Nursing Diagnosis “Ineffective Health Self-Management” [50]	Costa et al.	*Rev Bras Enferm* ^4^	Provides a framework for nurses to make clinical judgments regarding the diagnosis of ineffective health self-management among individuals living with HIV.

^1^ *Journal of Holistic Nursing*; ^2^ *British Journal of Nursing*; ^3^ *Journal of Research in Nursing*; ^4^ *Revista Brasileira de Enfermagem*; ^5^ *International Journal of Nursing Knowledge*; ^6^ *SAGE Open Nursing*; ^7^ *Research and theory for Nursing Practice*; ^8^ *Nursing Science Quarterly*. Thematic areas: [30,31,32,33,34,35,37,38,39,40,41,46,47]—clinical area; [28,29,36]—educative area; [42,43,44,45,48,49,50]—theoretical area.

## Data Availability

The datasets used and/or analyzed during the current study are available from the corresponding author on reasonable request. Additional data can be found in the journal itself.

## Public Involvement Statement

No public involvement in any aspect of this research.

## Guidelines and Standards Statement

This manuscript was prepared in accordance with the *Preferred Reporting Items for Systematic Reviews and Meta-Analyses Extension for Scoping Reviews* (PRISMA-ScR) [16].
